# Analysis of the Distribution and Antibiotic Resistance of Pathogens Causing Infections in Hospitals from 2017 to 2019

**DOI:** 10.1155/2022/3512582

**Published:** 2022-09-16

**Authors:** Guoliang Liu, Mingzhao Qin

**Affiliations:** Department of Geriatrics, Beijing Tongren Hospital, Capital Medical University, Beijing 100730, China

## Abstract

**Background:**

Antibiotic resistance is a global public health problem, leading to high mortality and treatment costs. To achieve more efficient treatment protocols and better patient recovery, the distribution and drug resistance of pathogens in our hospital were investigated, allowing significant clinical guidance for the use of antimicrobials.

**Methods:**

In this retrospective study (2017–2019), 3482 positive samples were isolated from 43,981 specimens in 2017; 3750 positive specimens were isolated from 42,923 specimens in 2018; and 3839 positive pathogens were isolated from 46,341 specimens in 2019. These samples were from various parts of the patients, including the respiratory tract, urine, blood, wound secretions, bile, and puncture fluids. The distribution and antibiotic resistance of these isolated pathogens from the whole hospital were analyzed.

**Results:**

The results from pathogen isolation showed that *Escherichia coli* (12.8%), *Staphylococcus aureus* (11%), *Klebsiella pneumoniae* (10.8%), *Pseudomonas aeruginosa* (10.7%), and *Acinetobacter baumannii* (6.4%) represented the five main pathogenic bacteria in our hospital. *Pseudomonas aeruginosa* (16.2% and 17.5%) occupied the largest proportion in the central intensive care unit (central ICU) and respiratory intensive care unit (RICU), while *Acinetobacter baumannii* (15.4%) was the most common pathogen in the emergency intensive care unit (EICU). The resistance rate of *Escherichia coli* to trimethoprim and minocycline was 100%, and the sensitivity rate to ertapenem, furantoin, and amikacin was above 90%. The resistance rate of *Pseudomonas aeruginosa* to all antibiotics, such as piperacillin and ciprofloxacin, was under 40%. The sensitivity rate of *Acinetobacter baumannii* to tigecycline and minocycline was less than 30%, and the resistance rate to many drugs such as piperacillin, ceftazidime, and imipenem was above 60%. Extended-spectrum *β*-lactamases (ESBLs)-producing *Klebsiella pneumoniae* (ESBLs-KPN) and carbapenem-resistant *Klebsiella pneumoniae* (CRE-KPN), ESBLs-producing *Escherichia coli* (ESBLs-ECO) and carbapenem-resistant *Escherichia coli* (CRE-ECO), multidrug-resistant *Acinetobacter baumannii* (MDR-AB), multidrug-resistant *Pseudomonas aeruginosa* (MDR-PAE), and methicillin-resistant *Staphylococcus aureus* (MRSA) are all important multidrug-resistant bacteria found in our hospital. The resistance rate of ESBLs-producing *Enterobacteriaceae* to ceftriaxone and amcarcillin-sulbactam was above 95%. CRE *Enterobacteriaceae* bacteria showed the highest resistance to amcarcillin-sulbactam (97.1%), and the resistance rates of MDR-AB to cefotaxime, cefepime, and aztreonam were 100%. The resistance rates of MDR-PAE to ceftazidime, imipenem, and levofloxacin were 100%, and the sensitivity rate to polymyxin B was above 98%. The resistance rate of MRSA to oxacillin was 100%, and the sensitivity rate to linezolid and vancomycin was 100%.

**Conclusion:**

The distribution of pathogenic bacteria in different hospital departments and sample sources was markedly different. Therefore, targeted prevention and control of key pathogenic bacteria in different hospital departments is necessary, and understanding both drug resistance and multiple drug resistance of the main pathogenic bacteria may provide guidance for the rational use of antibiotics in the clinic.

## 1. Introduction

Due to the complexity and universality of infectious diseases, antibacterial agents have been widely used in clinical practice. Since the application of antibacterial agents in clinical practice, they have saved the lives of countless patients. However, bacterial resistance caused by overuse not only has a negative impact on individual users but also on the social group as a whole. Globally, various institutes and agencies have recognized this serious public health issue. Antibiotics are a subset of antimicrobial agents that play a key role in the inhibition of essential bacterial functions and are used widely to treat and prevent bacterial infections in humans and other animals [1]. Treatment by antibiotics is one of the main approaches used by modern medicine to combat infectious diseases [2]. Antibiotics have not only saved countless lives but also have played a pivotal role in achieving significant advances in medicine and surgery and have successfully prevented or treated infections that occur in patients [3]. However, antibiotic resistance has emerged because of their overuse and inappropriate prescribing, as well as their extensive use in agriculture [4]. A minimum of 700,000 people die from antimicrobial-resistant infections each year around the world, and drug-resistant infections are expected to kill 10 million people a year within 30 years, greatly exceeding deaths from cancer. It has also been estimated that this resistance problem will be the biggest challenge facing healthcare systems by 2050 [1]. The rapid and sustained spread of antibiotic resistance poses a growing threat to the public, animal, and environmental health worldwide. The abuse of antibiotics in clinical practice, poor public health conditions, and insufficient public awareness are the main causes cited [5].

Multidrug resistance (MDR) relates to bacteria becoming resistant to multiple classes of antibiotics and [6, 7] is now classified as follows: multidrug resistance (MDR) that is not susceptible to at least one representative from each of the three categories of selected antimicrobial compound families [7]. Extreme drug resistance (XDR) is not susceptible to at least a single representative of all but very few categories of antimicrobial compounds. Pan-drug resistance (PDR) is not susceptible to any of the tested representatives of all known antimicrobial compound families [7]. Compared with other infections, MDR infections are associated with poorer clinical outcomes, resulting in increased morbidity and mortality rates and higher healthcare costs [8]. There is concern that the emergence of pan-resistant strains (pathogens resistant to all available antibiotics) will render some infections untreatable. How to effectively slow down the emergence of multidrug-resistant bacteria and block the spread of multidrug-resistant bacteria has attracted extensive attention from the medical community, government, and society.

In this study, the isolation, culture, and identification of pathogenic microorganisms and antimicrobial sensitivity tests were carried out, the detection results for different pathogenic microorganisms were provided, and the changes to and the mechanism of drug resistance were analyzed. This study provides a theoretical basis for exploring the clinical application of antibacterial drugs and further monitoring bacterial resistance and multidrug-resistant bacteria.

## 2. Samples and Methods

### 2.1. Source of Pathogenic Samples

Pathogen samples, including sputum, mid-section urine, blood, wound secretions, chest and gastric juices, bile, and puncture fluids, were taken from hospitalized patients from 2017 to 2019. To avoid overestimating antibiotic resistance, duplicate strains obtained from the same patient were deleted from the study. The study protocol was approved by the Ethics Committee of our hospital and given that medical records and patient information were anonymously reviewed and collected in this observational study, informed consent was not needed.

In 2017, the total number of microbial culture samples submitted for inspection was 43,981, and the top five infection sites were the lower respiratory tract (271/28.65%), urinary tract (125/13.21%), upper respiratory tract (107/11.31%), eyes, ears, and oral cavities (67/7.08%), and blood (64/6.77%). Respiratory tract infection, however, has always represented the main site of infection.

In 2018, the total number of microbial culture samples submitted for inspection was 42,923, a slight decrease from last year. The respiratory tract, urine, blood, stool, and female reproductive tract samples ranked in the top five, of which the respiratory tract samples, urine specimens, and blood specimens accounted for 43.93%, 12.35%, and 9.98% of the total, respectively. Stool specimens accounted for 6.73%, and female reproductive tract specimens accounted for 6.12%, a significant increase from last year by 4% and were related to *Streptococcus agalactiae* screening in obstetrics and gynecology.

The total number of microbial culture specimens submitted for inspection in 2019 was 46,341, also representing an increase from last year. The lower respiratory tract, urine, and blood specimens ranked in the top three, accounting for 39.6%, 11.0%, and 8.8% of the total, respectively, and the female reproductive tract specimens accounted for 6.7%, an increase of 6.12% from 2018. The main reason is related to *Streptococcus agalactiae* screening in the obstetrics and gynecology department, and stool specimens accounted for 6.5% and were related to the decline in the number of intestinal outpatients in recent years.

### 2.2. Strain Isolation, Strain Identification, and Antimicrobial Susceptibility Testing

We isolated and identified bacteria using standard microbiological and biochemical methods. According to the clinical operation requirements of the National Clinical Inspection Operation Regulations (3rd Edition), various specimens were cultured and bacterial identification was performed using a Vitek 2 Company instrument and supporting identification cards with microbiological tubes. Extended-spectrum *β*-lactamases (ESBLs)-producing *Klebsiella pneumoniae* (ESBLs-KPN), ESBLs-producing *Escherichia coli* (ESBLs-ECO), carbapenem-resistant (CRE) *Klebsiella pneumoniae* (CRE-KPN), CRE *Escherichia coli* (CRE-ECO), multidrug-resistant *Acinetobacter baumannii* (MDR-AB), multidrug-resistant *Pseudomonas aeruginosa* (MDR-PAE), and methicillin-resistant *Staphylococcus aureus* (MRSA) were defined based on their resistance to all antimicrobial agents as reported previously [6].

In addition, instrument drug sensitivity cards and Kirby–Bauer agar diffusion methods were used to define antibiotic resistance. The results were interpreted according to the minimum inhibitory concentration (MIC) interpretive breakpoints recommended by the Clinical and Laboratory Standards Institute (CLSI) of 2016. The quality-control strains were *Escherichia coli* ATCC 25922, *Pseudomonas aeruginosa* ATCC 27853, *Klebsiella pneumoniae* ATCC 700603, *Acinetobacter baumannii* ATCC 19606, *Staphylococcus aureus* ATCC 25923, *Staphylococcus epidermidis* ATCC 13518, and *Enterococcus faecium* ATCC 29212.

### 2.3. Monitoring and Analysis of Multidrug-Resistant Bacteria

Our hospital microbiology laboratory uses special statistical software MDR for drug resistance analysis to conduct multidrug resistance analysis on the main pathogenic bacteria (*Enterobacteriaceae, Acinetobacter baumannii, Pseudomonas aeruginosa*, and *Staphylococcus aureus*). An interim standard definition of MDR, XDR, and PDR terms coauthored by experts from the United States, Israel, Greece, Switzerland, and Australia [6] was used to identify the drug resistance of the samples.

### 2.4. Statistical Analyses

Data from our study were analyzed with SPSS (version 22.0, IBM Corp., Armonk, NY) and Microsoft Excel software 2007 (Microsoft Corporation, Redmond, WA). Proportions were used to summarize categorical data as appropriate.

## 3. Results

### 3.1. Isolation of Pathogenic Bacteria

According to the results from the pathogen bacteria isolation from the three hospital departments from 2017 to 2019 (Table 1), the top five pathogenic bacteria in three years were always *Escherichia coli* (12.8%), *Staphylococcus aureus* (11%), *Klebsiella pneumoniae* (10.8%), *Pseudomonas aeruginosa* (10.7%), and *Acinetobacter baumannii* (6.4%), which were relatively concentrated, and accounted for 51%, 53.4%, and 50.7% of the total cases each year. The average share of *Enterococcus faecalis* and *Enterococcus faecium* was 7.1% within three years.

From 2017 to 2019, the results of pathogenic bacterial isolation in the central intensive care unit (central ICU), respiratory intensive care unit (RICU), and emergency intensive care unit (EICU) were surveyed. Within the three ICU departments, *Escherichia coli*, *Staphylococcus aureus*, *Klebsiella pneumoniae*, *Pseudomonas aeruginosa,* and *Acinetobacter baumannii* were always in the top eight within the three years. In central ICU, *Pseudomonas aeruginosa* was ranked first over three years and had the highest proportion between 15.4% and 17.3%, followed by *Klebsiella pneumoniae* (36/13.7%), and both showed an upward trend from 2017 to 2019. *Acinetobacter baumannii* (24 strains) and *Escherichia coli* (18 strains) came in third and fourth, with a proportion of 9.5% and 6.8%, respectively. *Staphylococcus aureus* (17/6.5%) and *Enterococcus faecium* (15/5.9%) also consistently ranked in the top eight for three years (Table 2). In RICUs, six pathogenic bacteria always ranked in the top eight. *Pseudomonas aeruginosa* (17/17.5%) had the largest average share over three years among the three ICU departments. The next was *Klebsiella pneumoniae* (14/14.8%), *Escherichia coli* (11/11%), *Acinetobacter baumannii* (7/7.7%), and *Staphylococcus aureus* (6/6.3%). In addition, *Stenotrophomonas maltophilia* (9 strains) accounted for 9.1%, and the average proportion was highest in the three ICU departments (Table 3). In EICUs, *Acinetobacter baumannii* (14/15.4%) had the highest average proportion from 2017 to 2019, followed by *Klebsiella pneumoniae* (14/14.7%), which ranked second for three consecutive years. *Pseudomonas aeruginosa* (13/13.7%), *Escherichia coli* (12/13.4%), *Enterococcus faecium* (8/8.1%), *Staphylococcus aureus* (7/7.1%), and *Corynebacterium striatum* (6/6.7%) are also consistently ranked in the top eight for three years and their average proportion was highest in the three ICU departments, respectively (Table 4).

### 3.2. Distribution of Isolated Strains from Blood, Urine, and Sputum Samples

The composition of isolates from different sources from 2017 to 2019 was analyzed, and the results are shown in Tables 5–7. From 2017 to 2019, the average proportion of *Escherichia coli* isolates (61/22.8%) in blood samples was the highest, showing a downward trend. At the same time, *Staphylococcus epidermidis* (48/18.1%) and *Klebsiella pneumoniae* (32/12%) occupied the second and third places in each of the three years. The mean proportion of *Staphylococcus epidermidis* in blood specimens was higher than that seen in urine within the three years, but it was not found in sputum specimens. The composition of blood samples in 2017 and 2019 ranked fourth and *Acinetobacter baumannii* accounted for about 6.7%, but *Staphylococcus hominis* ranked fourth in 2018, accounting for 7.5%, *Staphylococcus hominis* ranked fifth for the three years, accounting for 8.1%, and was unique to blood samples (Table 5).

It was found that *Escherichia coli* (39.7%), *Enterococcus faecium* (11.3%), *Enterococcus faecalis* (9.4%), and *Klebsiella pneumoniae* (9.1%) ranked in the top four pathogenic bacteria from urine sample isolates. The most predominant pathogen in the urine samples was *Escherichia coli* accounting for 42.2%, 39.3%, and 37.8% from 2017 to 2019. Within the three years, compared to the blood and sputum samples, *Escherichia coli* accounted for the highest proportion of the urine samples isolated strains. *Enterococcus faecium* and *Enterococcus faecalis* have a higher proportion in urine than in blood samples, and they were not present in samples (Table 6).

*Pseudomonas aeruginosa* (22.5%), *Klebsiella pneumoniae* (20.6%), and *Acinetobacter baumannii* (16.6%) were the top three in sputum sample isolated strains. *Staphylococcus aureus* (8.3%) and *Stenotrophomonas maltophilia* (6.1%) were also common in sputum specimens and ranked fourth and fifth. Moreover, *Stenotrophomonas maltophilia* is a pathogen specific to sputum samples, and its proportion was increasing from 5.2% to 7.7% during 2017 to 2019 (Table 7).

### 3.3. Antibiotic Resistance Analysis

Combining the isolation of the pathogenic bacteria from the three hospital departments from 2017 to 2019 and the distribution of isolated strains from blood, urine and sputum specimens, it can be seen that the bacteria that are susceptible and have a high titer in each specimen were mainly *Escherichia coli*, *Klebsiella pneumoniae*, *Pseudomonas aeruginosa*, *Staphylococcus aureus,* and *Acinetobacter baumannii* and their antibiotic resistance was found to be unchanged.

From 2017 to 2019, *Escherichia coli* was generally resistant to trimethoprim and minocycline, with a resistance rate of up to 100% and with high sensitivity to imipenem, amikacin, ertapenem, and other drugs (Table 8). The resistance rate of *Klebsiella pneumoniae* to trimethoprim, cefuroxime, piperacillin, piperacillin-sulbactam, and ampicillin was higher than 90%. However, its resistance to cefoperazone-sulbactam, ertapenem, and amikacin was lower than 40% (Table 9). The resistance of *Pseudomonas aeruginosa* to most antibiotics such as piperacillin, ciprofloxacin, amikacin, and tobramycin was less than 30%, and resistance to polymyxin B was less than 5%, and even reached a sensitivity of 100% in 2018 and 2019 (Table 10). *Acinetobacter baumannii* had high sensitivity to tigecycline and minocycline of less than 30%, and the resistance rate to tigecycline was zero but was greater than 60% resistant to many drugs such as piperacillin, ceftazidime, gentamicin, and imipenem (Table 11). *Staphylococcus aureus* had the highest resistance rate to penicillin, at more than 80%, and the resistance rate to erythromycin was approximately 60%. However, no strains were resistant to antibiotics such as vancomycin, teicoplanin, tigecycline, and linezolid (Table 12).

### 3.4. Multidrug Resistance Analysis

**Figure 1:**
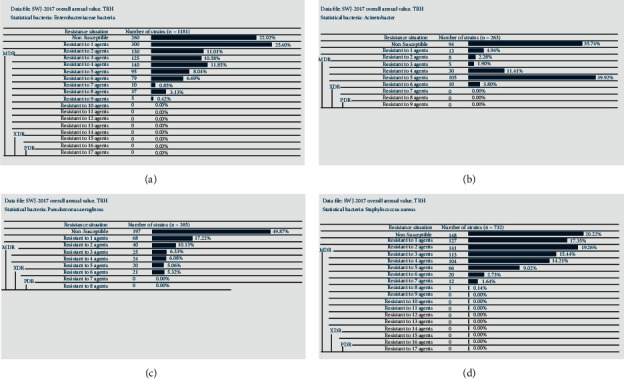
Analysis of multiple drug resistance for the main pathogenic bacteria in our hospital in 2017. (a) The analysis of multiple drug resistance of *Enterobacteriacea*e bacteria. (b) The analysis of multiple drug resistance of *Acinetobacter* bacteria. (c) The analysis of multiple drug resistance of *Pseudomonas aeruginosa*. (d) The analysis of multiple drug resistance of *Staphylococcus* bacteria.

Analysis of multiple drug resistance for the main pathogenic bacteria in our hospital in 2017 is shown in Figure 1. In 2017, a total of 1181 multidrug-resistant bacterial strains of *Enterobacteriacea*e were isolated, accounting for the largest proportion of the detected multidrug-resistant strains; of which 491 strains of multidrug-resistant organisms (MDRO) accounted for 41.6%, and no XDR and PDR strains were found (Figure 1(a)). ESBLs-KPN is highly resistant to amoxicillin and ceftriaxone, with resistance rates of 100% and 99.4%, respectively, and the sensitivity to ertapenem, imipenem, and piperacillin/tazobactam was above 95% (Table 13). The resistance rate of CRE-KPN to all drugs was above 50%, among which ampicillin, cefoperazone-sulbactam, ampicillin-sulbactam, ceftazidime, and ceftriaxone were all resistant by 100%. The resistance rates to nitrofurantoin, ciprofloxacin, levofloxacin, aztreonam, and cefepime were all greater than 95% (Table 14) and the resistance rates of ESBLs-producing *Escherichia coli* (ESBLs-ECO) to ampicillin and ceftriaxone were over 99%, and sensitivities to drugs such as amikacin, nitrofurantoin, and cefepime were all greater than 60%, with no strains being resistant to ertapenem, piperacillin-tazobactam, or imipenem (Table 15). A total of 263 strains of *Acinetobacter* were isolated, including 150 strains of MDRO, accounting for 57%, and no XDR and PDR strains were found (Figure 1(b)). The resistance rate of MDR-*Acinetobacter baumannii* (MDR-AB) to levofloxacin, moxifloxacin, and ampicillin was up to 100%, and the drug resistance to cotrimoxazole, amikacin, and other drugs was also more than 70% (Table 16). Of the 395 strains of *Pseudomonas aeruginosa* isolated, 90 strains of MDRO accounted for 22.8%, and 21 strains of XDR accounted for 5.3%. No PDR strain was found (Figure 1(c)). MDR-*Pseudomonas aeruginosa* (MDR-PAE) showed more than 97% resistance to ciprofloxacin, piperacillin, and amtronam, among which the resistance rate for ceftazidime, imipenem, and levofloxacin was 100%. While sensitivity to polymyxin B and tobramycin had a sensitivity of 98.7% (Table 17). A total of 732 strains of *Staphylococcus* were isolated, of which 316 were MDRO strains, accounting for 43.2%, and no XDR and PDR strains were found (Figure 1(d)). Methicillin-resistant *Staphylococcus aureus* (MRSA) was 100% resistant to benzacillin, 60% resistant to erythromycin, 50% resistant to ciprofloxacin, clindamycin, and tetracycline, but 100% sensitive to linezolid and vancomycin (Table 18).

**Figure 2:**
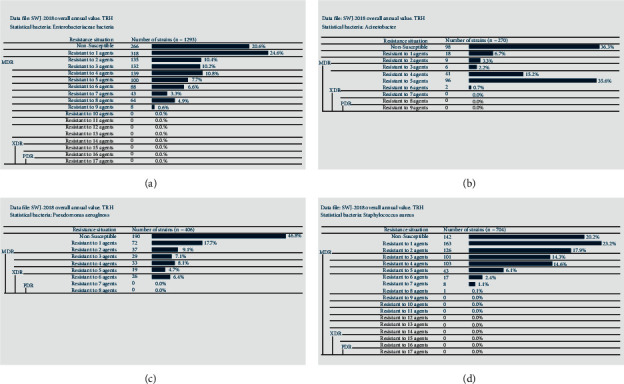
Analysis of multiple drug resistance for the main pathogenic bacteria in our hospital in 2018. (a) The analysis of multiple drug resistance of *Enterobacteriaceae* bacteria. (b) The analysis of multiple drug resistance of *Acinetobacter* bacteria. (c) The analysis of multiple drug resistance of *Pseudomonas aeruginosa*. (d) The analysis of multiple drug resistance of *Staphylococcus* bacteria.

In 2018, a total of 1293 strains of multidrug-resistant bacteria such as *Enterobacteriaceae* were isolated, of which MDRO (574 strains) accounted for 44.4%, while XDR and PDR strains were not found (Figure 2(a)). A total of 270 strains of *Acinetobacter* were isolated, including 145 strains of MDRO, accounting for 53.7%, and no XDR and PDR strains were found (Figure 2(b)). A total of 406 strains of *Pseudomonas aeruginosa* were isolated, among which 107 strains of MDRO accounted for 26.4%, while 26 strains of XDR accounted for 6.4%, and no PDR strains were found (Figure 2(c)). A total of 704 strains of *Staphylococcus* bacteria were isolated, including 300 strains (42.6%) of MDRO, with no XDR and PDR strains being found (Figure 2(d)). The resistance rates of MRSA to benzacillin and penicillin were 100% and 99.2%, respectively. No strains were found to be resistant to linezolid, vancomycin, teicoplanin, and tigecycline (Table 19).

**Figure 3:**
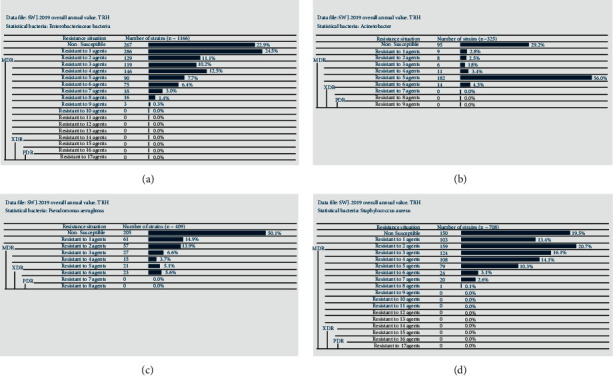
Analysis of multiple drug resistance for the main pathogenic bacteria in our hospital in 2019. (a) The analysis of multiple drug resistance of *Enterobacteriaceae* bacteria. (b) The analysis of multiple drug resistance of *Acinetobacter* bacteria. (c) The analysis of multiple drug resistance of *Pseudomonas aeruginosa*. (d) The analysis of multiple drug resistance of *Staphylococcus* bacteria.

As shown in Figure 3(a), in 2019, a total of 1166 strains of *Enterobacteriaceae* were isolated, of which 484 strains were isolated by MDR, accounting for 41.5%, and no XDR and PDR strains were found. The high resistance of ESBLs-producing *Enterobacteriaceae* to ceftriaxone and amcarcillin-sulbactam was observed, both more than 95%. Its drug resistance to cephalosporin, tobramycin, and furantoin was less than 40%, among which the drug resistance rate for tigecycline, imipenem, and amikacin was less than 5% (Table 20). Carbapenem-resistant (CRE) *Enterobacteriaceae* bacteria showed the highest resistance to amcarcillin-sulbactam (97.1%), and the resistance rate to most drugs ranged from 70% to 90%, but they were sensitive to tigecycline and amikacin (Table 21). A total of 325 strains of *Acinetobacter* were isolated, of which 213 strains were isolated from MDR, accounting for 65.5%, and no XDR and PDR strains were found (Figure 3(b)). A total of 409 strains of *Pseudomonas aeruginosa* were isolated, of which 86 strains were isolated by MDR, accounting for 21.0%, and 23 strains were isolated by XDR, accounting for 5.6%, with no PDR strain being found (Figure 3(c)). A total of 768 strains of *Staphylococcus* were isolated, of which 356 strains were isolated by MDRO, accounting for 46.4%, and no XDR and PDR strains were found (Figure 3(d)). Similar to 2018, MRSA showed 100% resistance to penicillin and benzacillin, and the sensitivity to tetracycline, ciprofloxacin, and other drugs was more than 60%, and no strains resistant to linezolid, vancomycin, and other four drugs were found (Table 22).

### 3.5. The Trend of Isolate Major Multidrug-Resistant Bacteria in Our Hospital in the Past Four Years

**Figure 4:**
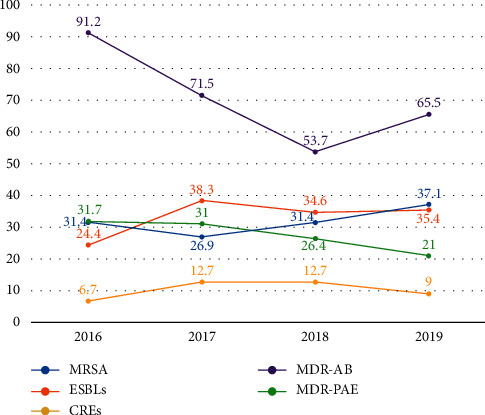
The trend of separation rate (%) of main multidrug-resistant strains in our hospital in recent four years.

As shown in Figure 4, the isolation rate of MDR-AB, which remained at the top for three years, declined in 2018 but increased again in 2019. ESBLs-ranked second in the three-year average separation rate, while MDR-PAB showed a continuous downward trend, whereas MRSA was the opposite, with a continuous increase being observed and CRE also exhibited a rise.

## 4. Discussion

The discovery of antibiotics in the last century is considered one of the most important achievements in the history of medicine, and its use has greatly reduced morbidity and mortality associated with bacterial infections [2]. However, the evolution of new bacterial strains, as well as the excessive use and reckless consumption of antibiotics, has led to the development of antibiotic resistance. Multidrug resistance is a potential threat worldwide and is escalating at an extremely high rate [9]. Poor public health conditions, lack of awareness concerning drug-resistant bacteria among the public, high incidences of disease, ease of access, and their misuse are the major factors exacerbating the problem [5]. In the context of antibiotic resistance, due to the emergence and increased prevalence of multidrug-resistant (MDR) superbugs such as *Staphylococcus aureus*, *Escherichia coli*, and *Klebsiella pneumoniae*, human health is being treated as a priority for the health of interdependent animals and related environments and is estimated to impose a significant health burden on the global population [10]. Therefore, we identified the clinical isolates obtained in the hospital from 2017 to 2019, carried out drug susceptibility tests and epidemiological infection analysis, obtained information about the pathogens for the whole hospital, and conducted a summary analysis, hoping to promote the rational use of antibiotics and play an active role in reducing the emergence of resistant bacteria in hospitals and controlling the spread of multidrug-resistant strains.

From 2017 to 2019, the isolation of pathogenic bacteria in the three departments of the hospital showed that the top five pathogens remained unchanged. These included *Escherichia coli*, *Staphylococcus aureus*, *Pseudomonas aeruginosa*, *Klebsiella pneumoniae,* and *Acinetobacter baumannii*, which, together with *Enterobacter faecium* as the most problematic clinical pathogens, were summarized as “ESKAPE” bugs by Louis Rice [11]. ESKAPEE pathogens have developed resistance mechanisms against most antibiotic treatments, including those that are the last line of defense, such as carbapenems and polymyxins [12]. According to the results of pathogen isolation in three ICU departments in the past three years, the five pathogens mentioned above always ranked among the top eight. The total number of isolates from central ICUs was always higher than that from specialized ICUs, namely RICUs and ICUs. The isolation rates of *Pseudomonas aeruginosa*, *Klebsiella pneumoniae,* and *Stenotrophomonas maltophilia* in the RICUs were the highest among the three ICU wards because they were all closely associated with lower respiratory tract infections [13]. In the last three years, the average proportion of *Pseudomonas aeruginosa* isolates was 17.5% in RICUs, similar to studies in the United States during the early years that found *P. aeruginosa* (17.0%) as a relatively common organism isolated in RICU with respiratory infections [14]. In EICUs, *Acinetobacter baumannii* occupies the highest isolation rate among the three ICU wards, and critically ill patients tend to be more susceptible to infection. Because *Acinetobacter baumannii* infection is associated with invasive surgery, the reason for hospitalization includes host factors, length of ICU stay, and prior use of broad-spectrum antibiotics [15].

The composition of isolates from different sources from 2017 to 2019 was analyzed, and we found that the isolation rate of *Staphylococcus epidermidis* was higher in blood samples than in urine samples, but no isolates were found in sputum samples. *Staphylococcus hominis* isolates were only present in blood samples, and as previously reported, these two bacteria both produce biofilms that allow them to adhere to internal medical devices and are commonly isolated from bloodstream infections [16, 17]. Among the three sources, blood, urine, and sputum, *Escherichia coli* isolates accounted for the highest proportion in urine specimens. *Enterococcus faecium* and *Enterococcus faecalis* were distributed at higher levels in urine samples than in blood samples and were absent in sputum samples. As previously reported, the above three bacteria are the main pathogenic bacteria of urinary tract infections [18, 19]. The top five frequent isolates from sputum samples are *Pseudomonas aeruginosa*, *Klebsiella pneumoniae*, *Acinetobacter baumannii*, *Staphylococcus aureus,* and *Stenotrophomonas maltophilia*, and this is similar to previous findings [13].

Measures for the management and clinical application of antibiotics in China are as follows: according to the notice of the Health and Family Planning Commission of the People's Republic of China on further strengthening the management of the clinical application of antibacterial drugs to effectively curb bacterial resistance, medical institutions should carry out monitoring of bacterial resistance, establish bacterial resistance early warning mechanisms, and take the following corresponding measures: (1) If the antimicrobial drug resistance rate of the main target bacteria exceeds 30%, warning information should be reported to the medical staff of the institution in a timely manner; (2) Antibiotics with a resistance rate of more than 40% for the major target bacteria should be used cautiously and empirically; (3) Antibiotics with drug resistance rates of over 50% for the major target bacteria should be selected according to drug sensitivity test results; (4) Clinical application of antibacterial drugs with drug resistance rates exceeding 75% for the main target bacteria should be suspended, and clinical application should be decided according to results based on bacterial resistance.

Regarding antibiotic resistance, *Escherichia coli* showed low resistance to most third-generation cephalosporins and aminoglycoside antibiotics, the resistance rate is between 30% and 50%, which is similar to the study conducted by Miller et al. [20]. It is highly sensitive to imipenem, nitrofurantoin, piperacillin-tazobactam, and amikacin and is recommended for clinical use. *Klebsiella pneumoniae*, also belonging to the *Enterobacteriaceae* family, exhibited low resistance to imipenem and cefoperazone-sulbactam. Similar antibiotic resistance rates have been reported by Liu et al. [21]. In 2018-2019, its resistance rate to amikacin, piperacillin-tazobactam, ertapenem, and other antibacterial drugs was less than 20%, indicating a wide range of drug choices that can be used as a good choice for current clinical treatment. *Pseudomonas aeruginosa* showed low to moderate rates of drug resistance to commonly used antipseudomonal drugs and most antibiotics such as carbapenems, amikacin, cefoperazone-sulbactam, piperacillin-tazobactam, and ceftazidime, were less than 30%, similar to the results of previous studies [22]. Thus, there are many options for medication. Especially in 2018 and 2019, no strains resistant to polymyxin B were found, and therefore, it is the recommended drug for clinical treatment. The drug resistance of *Acinetobacter baumannii* is relatively serious, and the resistance rate to most antibiotics is greater than 60%. Therefore, carbapenems are not recommended for single *Acinetobacter baumannii* infections, which can easily increase the risk of multidrug resistance. *Acinetobacter baumannii* has relatively high sensitivity to cefoperazone-sulbactam, which is the first choice for empirical medication in confirmed cases of infection to improve the curative effect. *Staphylococcus aureus* is resistant to penicillin by more than 85%, so the clinical application for these target bacteria should be suspended. No resistant strains were found to linezolid, vancomycin, teicoranin, and tigecycline. Hence they represent a good choice for empirical treatment.

From 2017 to 2019, the important multidrug-resistant bacteria in our hospital included extended-spectrum *β*-lactamases (ESBLs)-producing *Klebsiella pneumoniae* (ESBLs-KPN) and carbapenem-resistant *Klebsiella pneumoniae* (CRE-KPN), ESBLs-producing *Escherichia coli* (ESBLs-ECO) and carbapenem-resistant *Escherichia coli* (CRE-ECO), multidrug-resistant *Acinetobacter baumannii* (MDR-AB), multidrug-resistant *Pseudomonas aeruginosa* (MDR-PAE), and methicillin-resistant *Staphylococcus aureus* (MRSA), which were mainly detected by Chinese Antimicrobial Resistance Surveillance System.

*Acinetobacter baumannii*, Enterobacteriaceae, and *Pseudomonas aeruginosa* are the common clinical carbapenem-resistant Gram-negative bacteria. Several drugs that are active against carbapenem-resistant *Acinetobacter baumannii* have been approved for clinical use or have entered late-stage clinical development, including eravacycline, cefiderocol, and plazomicin [23]. For MDR-AB, carbapenems are not recommended for empirical use, not only because of their high resistance rate, but more importantly, they further increase the risk of multidrug resistance caused by high intensity antimicrobial use. For pan-resistant *Acinetobacter baumannii*, some clinical departments have chosen tigecycline for treatment, but CLSI (American Institute of Clinical and Laboratory Standards) lacks the criteria for determining the susceptibility of *Acinetobacter baumannii* to tigecycline, and its efficacy remains to be validated.

The detection rate of multidrug-resistant bacteria in the *Enterobacteriaceae* family was the highest and was mainly concentrated on the detection of ESBLs-ECO, ESBLs-KPN, CRE-KPN, and CRE-ECO. The number of ESBLs-KPN and CRE-KPN isolates ranked first in 2017, followed by MDR-AB, and these results are in agreement with those obtained by Talaat et al. [24], who showed that the most predominant Gram-rods in the hospital were *Klebsiella pneumoniae* (28.7%) and Acinetobacter sp. (13.7%). ESBLs-producing isolates showed resistance to *β*-lactam antibiotics, including third-generation cephalosporins; in addition, they often exhibit resistance to other classes of drugs such as aminoglycosides, cotrimoxazole, and fluoroquinolones [25]. Tigecycline and imipenem can be used as empirical drugs for ESBL-producing bacteria. It should be emphasized that ESBLs-ECO and ESBLs-KPN have high drug resistance rates to ceftriaxone and amcarcillin-sulbactam, and the risk of induced drug resistance is also very high. Therefore, the drug sensitivity test results should be referred to for selection. The detection rate of CRE bacteria in 2019 was higher than the national average in 2018, and therefore, it is necessary to reduce the overuse of carbapenem antibiotics and prevent the spread of bacteria in hospitals and regions. The resistance rate of CRE bacteria to amcarcillin-sulbactam exceeded 95%, and their clinical use should be suspended. No strains sensitive to tigecycline have been found, and they can be used as clinically recommended drugs, usually in combination with other drugs. *Enterobacteriaceae* represents a key family of carbapenem-resistant bacteria. Colistin, tigecycline, ceftazidime-avibactam, plazomicin, eravacycline, and cefiderocol can all be used for their clinical treatment [23].

The average separation rate of MDR-PAE ranks third (31.7%), with no major fluctuations in recent years. It is also a common clinical carbapenem-resistant Gram-negative bacterium. Our results showed that MDR-PAE and XDR-PAE occupy 23.4% and 5.8% of the average proportion of *Pseudomonas aeruginosa* isolates, higher than the results from other studies. In 2015, the European Centers for Disease Prevention and Control stated that MDR-PAE and XDR-PAE isolates accounted for 13.7% and 5.5% [26]. The high prevalence of resistant species in developing countries could be due to noncompliance with infection control regulations and to the lack of or an imperfect antibiotic policy. Studies [26] have shown that multiple antibiotic combinations can be used as a clinical solution for MDR-PAE and XDR-PAE infections. Previous studies [27, 28] have reported that combinations of polymyxins with these anti-*pseudomonas* drugs (such as imipenem, piperacillin, aztreonam, ceftazidime, or ciprofloxacin) are more effective than polymyxins alone against MDR-PAE, providing a reference for the treatment of MDR-PAE infection. Yadav et al. [29] demonstrated substantially enhanced death *in vivo* against an MDR-PAE clinical isolate with an optimized imipenem-plus-tobramycin combination regimen, which was an alternative to colistin therapy, especially in patients with renal insufficiency. In addition, drugs such as cefiderocol and fosfomycin are potential treatment options in the near future [26]. The available clinical solution for MDR-PAE infections requires a precise diagnostic and combination antibiotic therapy based on diagnostics. Several infections which are recurrent need additional care to stop the proliferation of MDR-PAE contaminating the surrounding environment.

MRSA is a virulent and difficult-to-treat “superbug,” and our results show that MRSA accounted for 30% to 50% of *Staphylococcus aureus* infections in hospital settings over the three-year period, which was slightly higher than the 25% to 50% reported in previous studies [30]. As previously reported [31], the infection rates of resistant *Staphylococcus*, *Pseudomonas*, *Acinetobacter*, and *Klebsiella* vary by country and region, with Asia being higher than North America and Western Europe. This may be due to the apparent wide variations in health care systems, ICU facilities, and policies for infectious disease control in the different geographical regions. Drug resistance, however, is consistent with previous research results, where MRSA is resistant to penicillin-like beta-lactam antibiotics [32], and the resistance to penicillin was observed to be as high as 99.2%, and clinical use of this target bacterium should be suspended. Many drugs remain active against MRSA, including glycopeptides (vancomycin and teicoranin), linezolid, and tigecycline, to which no resistant strains have been found and are, therefore, good choices for empirical treatment. Even some newer lactams, such as ceftazlorin and cefdipropanol, can be used as treatment options for MRSA [33].

With the promotion of rational applications for antibiotics, the isolation spectrum of pathogenic bacteria and the isolation rate of multidrug-resistant strains in our hospital have also changed accordingly, mainly reflected by the fact that although the isolation and drug resistance rates of MDR-AB always ranked first. After 2016, the separation rate of MDR-AB decreased significantly, which is probably due to the implementation of the Guiding Principles of Clinical Use of Antibiotics in 2015. The prevalence of CRE *Enterobacteriaceae* bacteria has increased in recent years, which is consistent with the national drug resistance monitoring information. The isolation rates of other bacteria did not fluctuate greatly, but the epidemiology of these bacteria still needs to be addressed.

The emergence of multidrug-resistant bacteria, or superbugs, poses a serious threat to public health and requires multilevel efforts to prevent them from overcoming antibiotic resistance. Governments must allocate sufficient funds to improve and develop new drug products, monitor the use of antibiotics, and establish strict policies and regulations. In addition, infection control measures must be strictly implemented in hospitals, but management practices must be considered for the use of antibiotics and microbicides and appropriate disposal or discharge of medical waste. Clinicians should avoid prescribing unnecessary and excessive antibiotics to patients with normal infections and advise patients to follow good hygiene practices such as hand washing and appropriate infection control measures. As an individual, we can take antibiotics that are prescribed only by our doctors, take them exactly as prescribed, and use them sensibly. Efforts to address the spread of antibiotic resistance include limiting the overuse of antibiotics in the food and animal sectors.

Nonantibiotic strategies for the treatment of antibiotic-resistant pathogens have been reported, such as gene editing techniques, immunotherapies, and vaccines, and antivirulence inhibitor bacteriophages [5, 10]. Antimicrobial adjuvants, fecal microbiota transplant (FMT), and competitive exclusion of pathogens through genetically modified probiotics and postbiotics are prospective alternative, unconventional strategies [5]. In addition, epidemiological and surveillance studies should be carried out and powerful tools should be used to deepen our understanding of antibiotic resistance and provide a timely and precise diagnosis of antibiotic use and consumption. Therefore, a multidisciplinary approach is needed to eliminate the serious threat of multidrug resistance.

However, this study also has some limitations. When analyzing multiple drug resistance, multiple bacteria in the same family and genus were not studied separately. In the future, a specific analysis should be carried out for important multidrug-resistant pathogens.

## 5. Conclusion

The distribution of pathogenic bacteria in different hospital departments and sample sources is variable. Therefore, targeted prevention and control of key pathogenic bacteria in different hospital departments must be carried out. Understanding the drug resistance and multiple drug resistance of the main pathogenic bacteria can provide guidance for the rational use of antibiotics in clinic.

## Figures and Tables

**Table 1:** The top 15 isolated pathogens in the three districts of the hospital in 2017, 2018, and 2019.

Year	2017			2018			2019		
Rankings	Bacteria	Number	Proportion	Bacteria	Number	Proportion	Bacteria	Number	Proportion (%)
1	*Escherichia coli*	456	0.131	*Escherichia coli*	497	0.133	*Escherichia coli*	465	0.121
2	*Staphylococcus aureus*	384	0.11	*Klebsiella pneumoniae*	462	0.123	*Staphylococcus aureus*	410	0.107
3	*Pseudomonas aeruginosa*	370	0.106	*Staphylococcus aureus*	420	0.112	*Pseudomonas aeruginosa*	409	0.107
4	*Klebsiella pneumoniae*	356	0.102	*Pseudomonas aeruginosa*	406	0.108	*Klebsiella pneumoniae*	382	0.1
5	*Acinetobacter baumannii*	212	0.061	*Acinetobacter baumannii*	219	0.058	*Acinetobacter baumannii*	282	0.073
6	*Enterococcus faecalis*	156	0.045	*Staphylococcus epidermidis*	176	0.047	*Staphylococcus epidermidis*	251	0.065
7	*Vibrio parahaemolyticus*	135	0.039	*Enterococcus faecalis*	147	0.039	*Enterococcus faecium*	149	0.039
8	*Staphylococcus epidermidis*	129	0.037	*Enterococcus faecium*	120	0.032	*Stenostomonas maltophilia*	129	0.034
9	*Stenostomonas maltophilia*	103	0.03	*Streptococcus agalactiae*	116	0.031	*Streptococcus agalactiae*	123	0.032
10	*Streptococcus agalactiae*	99	0.028	*Enterobacter cloacae*	113	0.03	*Enterococcus faecalis*	122	0.032
11	*Enterobacter cloacae*	92	0.026	*Stenostomonas maltophilia*	87	0.023	*Enterobacter cloacae*	101	0.026
12	*Enterococcus faecium*	92	0.026	*Corynebacterium striatum*	84	0.022	*Haemophilus influenzae*	69	0.018
13	*Corynebacterium striatum*	68	0.02	*Streptococcus pneumoniae*	68	0.018	*Corynebacterium striatum*	68	0.018
14	*Streptococcus pneumoniae*	59	0.017	*Vibrio parahaemolyticus*	60	0.016	*Streptococcus pneumoniae*	64	0.017
15	*Proteus mirabilis*	58	0.017	*Proteus mirabilis*	52	0.014	*Streptococcus astragali*	53	0.014
	Other bacteria	714	0.205	Other bacteria	723	0.193	Other bacteria	762	0.198
	Total	3483	1	Total	3750	1	Total	3839	1

**Table 2:** Isolation of pathogenic bacteria in central intensive care units (central ICUs) in 2017, 2018, and 2019 years.

2017			2018			2019		
Bacteria	Number	Proportion	Bacteria	Number	Proportion	Bacteria	Number	Proportion
*Pseudomonas aeruginosa*	40	0.154	*Pseudomonas aeruginosa*	48	0.160	*Pseudomonas aeruginosa*	39	0.173
*Klebsiella pneumoniae*	32	0.123	*Klebsiella pneumoniae*	41	0.137	*Klebsiella pneumoniae*	34	0.150
*Acinetobacter baumannii*	26	0.100	*Escherichia coli*	29	0.097	*Acinetobacter baumannii*	25	0.111
*Burkholderia cepacia*	19	0.073	*Acinetobacter baumannii*	22	0.073	*Staphylococcus aureus*	19	0.084
*Staphylococcus aureus*	16	0.062	*Enterococcus faecium*	17	0.057	*Burkholderia cepacia*	17	0.075
*Escherichia coli*	14	0.054	*Enterococcus faecalis*	16	0.053	*Stenostomonas maltophilia*	15	0.066
*Enterococcus faecium*	14	0.054	*Staphylococcus aureus*	15	0.050	*Enterococcus faecium*	15	0.066
*Enterobacter cloacae*	12	0.046	*Enterobacter cloacae*	14	0.047	*Escherichia coli*	12	0.053
*Enterococcus faecalis*	10	0.038	*Staphylococcus epidermidis*	14	0.047	*Enterobacter cloacae*	10	0.044
*Staphylococcus epidermidis*	8	0.031	*Burkholderia cepacia*	9	0.030	*Staphylococcus epidermidis*	8	0.035
*Stenostomonas maltophilia*	8	0.031	*Corynebacterium striatum*	9	0.030	*Streptococcus pneumoniae*	8	0.035
*Corynebacterium striatum*	8	0.031	*Stenostomonas maltophilia*	9	0.030	*Enterococcus faecalis*	8	0.035
*Streptococcus pneumoniae*	6	0.023	*Haemophilus influenzae*	7	0.023	*Corynebacterium striatum*	6	0.027
*Klebsiella aerogenes*	6	0.023	*Klebsiella aerogenes*	7	0.023	*Klebsiella aerogenes*	6	0.027
Other bacteria	41	0.158	Other bacteria	43	0.143	Other bacteria	4	0.018
Total	260	1.000	Total	300	1.000	Total	226	1.000

**Table 3:** Isolation of pathogenic bacteria in respiratory intensive care units (RICUs) in 2017, 2018, and 2019.

2017			2018			2019		
Bacteria	Number	Proportion	Bacteria	Number	Proportion	Bacteria	Number	Proportion
*Escherichia coli*	18	0.176	*Pseudomonas aeruginosa*	22	0.204	*Pseudomonas aeruginosa*	14	0.184
*Pseudomonas aeruginosa*	14	0.137	*Klebsiella pneumoniae*	17	0.157	*Klebsiella pneumoniae*	12	0.158
*Klebsiella pneumoniae*	13	0.127	*Stenostomonas maltophilia*	11	0.102	*Stenostomonas maltophilia*	7	0.092
*Stenostomonas maltophilia*	8	0.078	*Corynebacterium striatum*	9	0.083	*Acinetobacter baumannii*	6	0.079
*Acinetobacter baumannii*	8	0.078	*Acinetobacter baumannii*	8	0.074	*Escherichia coli*	6	0.079
*Staphylococcus aureus*	7	0.069	*Burkholderia cepacia*	8	0.074	*Staphylococcus aureus*	5	0.066
*Staphylococcus epidermidis*	7	0.069	*Escherichia coli*	8	0.074	*Burkholderia cepacia*	4	0.053
*Enterococcus faecalis*	6	0.059	*Staphylococcus aureus*	6	0.056	*Morganella morganii*	3	0.039
*Proteus mirabilis*	4	0.039	*Proteus mirabilis*	4	0.037	*Staphylococcus epidermidis*	3	0.039
*Enterobacter cloacae*	3	0.029	*Enterobacter cloacae*	2	0.019	*Proteus mirabilis*	3	0.039
*Streptococcus pneumoniae*	2	0.020	*Enterococcus faecalis*	2	0.019	*Staphylococcus hominis*	2	0.026
*Corynebacterium striatum*	2	0.020	*Enterococcus faecium*	2	0.019	*Corynebacterium striatum*	2	0.026
*Staphylococcus capitis*	2	0.020	*Streptococcus pneumoniae*	2	0.019	*Enterobacter cloacae*	2	0.026
*Enterococcus faecium*	1	0.010	*Staphylococcus capitis*	1	0.009	*Enterococcus faecalis*	2	0.026
Other bacteria	7	0.069	Other bacteria	6	0.056	Other bacteria	5	0.066
Total	102	1.000	Total	108	1.000	Total	76	0.704

**Table 4:** Isolation of pathogenic bacteria in emergency intensive care units (EICUs) in 2017, 2018, and 2019.

2017			2018			2019		
Bacteria	Number	Proportion	Bacteria	Number	Proportion	Bacteria	Number	Proportion
*Pseudomonas aeruginosa*	15	0.140	*Acinetobacter baumannii*	19	0.200	*Escherichia coli*	13	0.163
*Klebsiella pneumoniae*	13	0.121	*Klebsiella pneumoniae*	16	0.168	*Klebsiella pneumoniae*	12	0.150
*Acinetobacter baumannii*	12	0.112	*Pseudomonas aeruginosa*	15	0.158	*Acinetobacter baumannii*	12	0.150
*Escherichia coli*	12	0.112	*Escherichia coli*	12	0.126	*Pseudomonas aeruginosa*	9	0.113
*Enterococcus faecium*	10	0.093	*Enterococcus faecium*	6	0.063	*Enterococcus faecium*	7	0.088
*Staphylococcus aureus*	8	0.075	*Staphylococcus aureus*	6	0.063	*Staphylococcus aureus*	6	0.075
*Corynebacterium striatum*	8	0.075	*Corynebacterium striatum*	5	0.053	*Corynebacterium striatum*	6	0.075
*Stenostomonas maltophilia*	8	0.075	*Stenostomonas maltophilia*	4	0.042	*Stenostomonas maltophilia*	4	0.050
*Enterococcus faecalis*	6	0.056	*Proteus mirabilis*	3	0.032	*Enterococcus faecalis*	4	0.050
*Burkholderia cepacia*	4	0.037	*Burkholderia cepacia*	2	0.021	*Staphylococcus epidermidis*	2	0.025
*Proteus mirabilis*	2	0.019	*Enterococcus faecalis*	2	0.021	*Proteus mirabilis*	2	0.025
*Staphylococcus haemolyticus*	1	0.009	*Staphylococcus haemolyticus*	1	0.011	*Staphylococcus haemolyticus*	1	0.013
*Corynebacterium afermentans*	1	0.009	*Corynebacterium urealyticum*	1	0.011	*Staphylococcus capitis*	1	0.013
*Staphylococcus capitis*	1	0.009	*Enterobacter avium*	1	0.011	*Saprophytic staphylococcus*	1	0.013
Other bacteria	6	0.056	Other bacteria	2	0.021	Other bacteria	0	0.000
Total	107	1.000	Total	95	1.000	Total	80	1.000

**Table 5:** Composition of blood specimen isolates in 2017, 2018, and 2019 years.

2017			2018			2019		
Bacteria	Number	Proportion	Bacteria	Number	Proportion	Bacteria	Number	Proportion
*Escherichia coli*	62	0.238	*Escherichia coli*	70	0.228	*Escherichia coli*	50	0.218
*Staphylococcus epidermidis *	45	0.173	*Staphylococcus epidermidis*	57	0.186	*Staphylococcus epidermidis*	42	0.183
*Klebsiella pneumoniae*	30	0.115	*Klebsiella pneumoniae*	42	0.137	*Klebsiella pneumoniae*	25	0.109
*Acinetobacter baumannii*	20	0.077	*Staphylococcus hominis*	23	0.075	*Acinetobacter baumannii*	13	0.057
*Pseudomonas aeruginosa*	12	0.046	*Staphylococcus aureus*	13	0.042	*Staphylococcus hominis*	11	0.048
*Staphylococcus aureus*	11	0.042	*Enterococcus faecalis*	12	0.039	*Enterococcus faecium*	10	0.044
*Staphylococcus hominis*	10	0.038	*Acinetobacter baumannii*	11	0.036	*Staphylococcus aureus*	9	0.039
*Enterobacter cloacae*	9	0.035	*Pseudomonas aeruginosa*	7	0.023	*Staphylococcus haemolyticus*	9	0.039
*Enterococcus faecium*	8	0.031	*Enterobacter cloacae*	6	0.020	*Pseudomonas aeruginosa*	7	0.031
*Staphylococcus haemolyticus*	4	0.015	*Enterococcus faecium*	4	0.013	*Burkholderia cepacia*	3	0.013
Other bacteria	49	0.188	Other bacteria	62	0.202	Other bacteria	50	0.218
Total	260	1.000	Total	307	1.000	Total	229	1.000

**Table 6:** Composition of urine specimen isolates in 2017, 2018, and 2019.

2017			2018			2019		
Bacteria	Number	Proportion	Bacteria	Number	Proportion	Bacteria	Number	Proportion
*Escherichia coli*	258	0.422	*Escherichia coli*	262	0.393	*Escherichia coli*	265	0.377
*Klebsiella pneumoniae*	75	0.123	*Enterococcus faecium*	69	0.103	*Enterococcus faecium*	86	0.123
*Enterococcus faecium*	70	0.114	*Enterococcus faecalis*	63	0.094	*Enterococcus faecalis*	59	0.084
*Enterococcus faecalis*	63	0.103	*Klebsiella pneumoniae*	51	0.076	*Klebsiella pneumoniae*	51	0.073
*Pseudomonas aeruginosa*	34	0.056	*Pseudomonas aeruginosa*	34	0.051	*Pseudomonas aeruginosa*	34	0.048
*Staphylococcus epidermidis *	22	0.036	*Staphylococcus epidermidis*	21	0.031	*Staphylococcus epidermidis*	28	0.040
*Proteus mirabilis*	17	0.028	*Proteus mirabilis*	18	0.027	*Streptococcus agalactiae*	18	0.026
*Enterobacter cloacae*	15	0.025	*Streptococcus agalactiae*	16	0.024	*Proteus mirabilis*	15	0.021
*Streptococcus agalactiae*	14	0.023	*Morganella morganii*	12	0.018	*Acinetobacter haemolyticus*	14	0.020
*Acinetobacter haemolyticus*	11	0.018	*Corynebacterium glutamicum*	11	0.016	*Enterobacter cloacae*	12	0.017
Other bacteria	33	0.054	Other bacteria	110	0.165	Other bacteria	120	0.171
Total	612	1.000	Total	667	1.000	Total	702	1.000

**Table 7:** Composition of sputum specimen isolates in 2017, 2018, and 2019.

2017			2018			2019		
Bacteria	Number	Proportion	Bacteria	Number	Proportion	Bacteria	Number	Proportion
*Pseudomonas aeruginosa*	280	0.233	*Klebsiella pneumoniae*	286	0.224	*Pseudomonas aeruginosa*	295	0.220
*Klebsiella pneumoniae*	262	0.218	*Pseudomonas aeruginosa*	282	0.221	*Acinetobacter baumannii*	247	0.185
*Acinetobacter baumannii*	203	0.169	*Acinetobacter baumannii*	183	0.143	*Klebsiella pneumoniae*	234	0.175
*Staphylococcus aureus*	100	0.083	*Staphylococcus aureus*	110	0.086	*Staphylococcus aureus*	108	0.081
*Escherichia coli*	85	0.071	*Stenostomonas maltophilia*	71	0.056	*Stenostomonas maltophilia*	103	0.077
*Stenostomonas maltophilia*	62	0.052	*Escherichia coli*	60	0.047	*Escherichia coli*	59	0.044
*Corynebacterium striatum*	48	0.040	*Corynebacterium striatum*	50	0.039	*Enterobacter cloacae*	52	0.039
*Enterobacter cloacae*	41	0.034	*Enterobacter cloacae*	48	0.038	*Corynebacterium striatum*	42	0.031
*Streptococcus pneumoniae*	36	0.030	*Streptococcus pneumoniae*	33	0.026	*Haemophilus influenzae*	40	0.030
*Burkholderia cepacia*	29	0.024	*Burkholderia cepacia*	31	0.024	*Burkholderia cepacia*	30	0.022
Other bacteria	54	0.045	Other bacteria	123	0.096	Other bacteria	128	0.096
Total	1200	1.000	Total	1277	1.000	Total	1338	1.000

**Table 8:** Drug resistance rates of *Escherichia coli* from 2017 to 2019.

	2017		2018		2019	
*Escherichia coli*	Drugs	Drug resistance rate (%)	Drugs	Drug resistance rate (%)	Drugs	Drug resistance rate (%)
	Trimethoprim	100	Ampicillin-sulbactam	71.3	Ampicillin-sulbactam	76.8
	Minocycline	100	Ciprofloxacin	60.4	Cefuroxime	54.3
	Cefazolin	90.27	Levofloxacin	55.9	Ciprofloxacin	54.1
	Ampicillin	87.7	Ceftriaxone	55.2	Levofloxacin	49.9
	Ceftriaxone	78	Cotrimoxazole	49.5	Ceftriaxone	48.3
	Ciprofloxacin	78	Gentamicin	39.6	Cotrimoxazole	45.3
	Levofloxacin	73.2	Aztreonam	36.5	Gentamicin	34.9
	Ampicillin-sulbactam	65.9	Ceftazidime	26.4	Aztreonam	27.9
	Compound sulfadiazine	64	Cefepime	23.3	Ceftazidime	20.3
	Aztreonam	55.2	Tobramycin	14.3	Cefepime	17.4
	Piperacillin	50	Cefoperazone-sulbactam	7.9	Tobramycin	10.5
	Tobramycin	49.2	Fosfomycin	7	Fosfomycin	7.0
	Cefepime	36.4	Ertapenem	5.2	Cefoperazone-sulbactam	3.4
	Gentamicin	36.3	Piperacillin-tazobactam	4.6	Nitrofurantoin	2.5
	Cefotaxime	32.4	Imipenem	4	Piperacillin-tazobactam	2.4
	Ceftazidime	32	Nitrofurantoin	2.9	Amikacin	1.7
			Amikacin	1.4	Ertapenem	0.7
					Imipenem	0.6

**Table 9:** Drug resistance rates of *Klebsiella pneumoniae* from 2017 to 2019.

	2017		2018		2019	
	Drugs	Drug resistance rate (%)	Drugs	Drug resistance rate (%)	Drugs	Drug resistance rate (%)
*Klebsiella pneumoniae*	Trimethoprim	100	Ampicillin-sulbactam	71.7	Ampicillin-sulbactam	66.7
	Cefuroxime	100	Nitrofurantoin	34.8	Nitrofurantoin	33.9
	Piperacillin	100	Fosfomycin	34.1	Cotrimoxazole	29.6
	Piperacillin-sulbactam	100	Ceftriaxone	30	Aztreonam	26.7
	Ampicillin	96.3	Cotrimoxazole	27	Cotrimoxazole	23.8
	Cefazolin	81.7	Levofloxacin	25.6	Ciprofloxacin	22.2
	Nitrofurantoin	78.8	Aztreonam	25.2	Ceftazidime	20.9
	Fosfomycin	72.5	Ciprofloxacin	25.1	Levofloxacin	19.9
	Ampicillin-sulbactam	70.8	Ceftazidime	23.9	Gentamicin	18.9
	Ceftriaxone	65.7	Gentamicin	22.7	Cefepime	17.1
	Cefepime	53.5	Cefepime	22.2	Tobramycin	13.5
	Tobramycin	52.6	Tobramycin	19	Imipenem	11.8
	Aztreonam	51.3	Cefoperazone-sulbactam	18.9	Piperacillin-tazobactam	11.3
	Compound sulfadiazine	50.8	Imipenem	17.8	Cefoperazone-sulbactam	11.1
	Ceftazidime	50	Ertapenem	17.3	Ertapenem	8.8
	Cefotaxime	50	Piperacillin-tazobactam	17	Amikacin	6.0
	Ciprofloxacin	49.2	Amikacin	12.2	Tigecycline	0.0
	Cefoperazone	48.6				
	Levofloxacin	47.2				
	Gentamicin	46.1				
	Piperacillin-tazobactam	44.3				
	Imipenem	40.2				
	Cefoperazone-sulbactam	32.4				

**Table 10:** Drug resistance rates of *Pseudomonas aeruginosa* from 2017 to 2019.

	2017		2018		2019	
	Drugs	Drug resistance rate (%)	Drugs	Drug resistance rate (%)	Drugs	Drug resistance rate (%)
*Pseudomonas aeruginosa*	Aztreonam	37.2	Meropenem	22	Meropenem	18.4
	Cefepime	34	Aztreonam	18.8	Imipenem	15.8
	Imipenem	33.5	Levofloxacin	18.8	Levofloxacin	13.7
	Piperacillin	29.1	Cefepime	18.7	Aztreonam	13.4
	Meropenem	27.1	Imipenem	18.2	Gentamicin	12.2
	Gentamicin	25.2	Gentamicin	15.6	Cefepime	12.1
	Piperacillin-sulbactam	25.1	Ciprofloxacin	13.5	Piperacillin	11.6
	Ceftazidime	23.7	Piperacillin	12.8	Cefoperazone-sulbactam	9.2
	Levofloxacin	20	Ceftazidime	12.2	Ciprofloxacin	8.6
	Ciprofloxacin	18.4	Cefoperazone-sulbactam	11.1	Tobramycin	7.7
	Tobramycin	13.5	Tobramycin	9.9	Piperacillin-tazobactam	7.2
	Amikacin	10	Amikacin	9.2	Ceftazidime	6.8
	Polymyxin B	2.5	Piperacillin-tazobactam	8.6	Amikacin	3.5
			Polymyxin B	0	Polymyxin B	0.0

**Table 11:** Drug resistance rates of *Acinetobacter baumannii* from 2017 to 2019.

	2017		2018		2019	
	Drugs	Drug resistance rate (%)	Drugs	Drug resistance rate (%)	Drugs	Drug resistance rate (%)
*Acinetobacter baumannii*	Piperacillin	73.5	Piperacillin	63	Piperacillin	73.2
	Moxifloxacin	74.7	Moxifloxacin	63.8	Imipenem	72.2
	Cefepime	73.6	Cefepime	63.7	Piperacillin-tazobactam	71.2
	Piperacillin-tazobactam	74.6	Piperacillin-tazobactam	63.6	Cefepime	70.0
	Ceftazidime	73.9	Ceftazidime	63.6	Ceftazidime	69.8
	Imipenem	73.1	Imipenem	62.7	Gentamicin	69.6
	Levofloxacin	72.5	Levofloxacin	62.6	Ciprofloxacin	67.7
	Gentamicin	69.7	Gentamicin	60.6	Levofloxacin	61.5
	Amikacin	66.5	Amikacin	58.1	Tobramycin	55.4
	Tobramycin	65.3	Tobramycin	57.2	Amikacin	42.2
	Cefoperazone-sulbactam	37.3	Cefoperazone-sulbactam	32.9	Minocycline	27.3
	Minocycline	25.6	Minocycline	21.5	Tigecycline	0.0
	Tigecycline	0	Tigecycline	0		

**Table 12:** Drug resistance rates of *Staphylococcus aureus* from 2017 to 2019.

	2017		2018		2019	
	Drugs	Drug resistance rate (%)	Drugs	Drug resistance rate (%)	Drugs	Drug resistance rate (%)
*Staphylococcus aureus*	Penicillin	91.3	Penicillin	87.6	Penicillin	89.6
	Erythromycin	61.8	Erythromycin	59.8	Erythromycin	62.7
	Clindamycin	58.6	Clindamycin	57.1	Clindamycin	58.4
	Oxacillin	35.2	Oxacillin	32.3	Oxacillin	36.8
	Tetracycline	24.2	Tetracycline	23.2	Cotrimoxazole	24.1
	Cotrimoxazole	17.3	Cotrimoxazole	16.3	Tetracycline	18.3
	Ciprofloxacin	16.5	Ciprofloxacin	15	Ciprofloxacin	18.0
	Gentamicin	14.8	Gentamicin	14	Moxifloxacin	15.7
	Moxifloxacin	14	Moxifloxacin	13.3	Levofloxacin	14.2
	Levofloxacin	13	Levofloxacin	10	Gentamicin	14.1
	Rifampicin	3.5	Rifampicin	3.3	Rifampicin	3.7
	Nitrofurantoin	1.2	Nitrofurantoin	0.8	Nitrofurantoin	0.8
	Linezolid	0	Linezolid	0	Linezolid	0.0
	Vancomycin	0	Vancomycin	0	Vancomycin	0.0
	Teicoplanin	0	Teicoplanin	0	Teicoplanin	0.0
	Tigecycline	0	Tigecycline	0	Tigecycline	0.0

**Table 13:** Analysis of multiple drug resistance rate of ESBLs-KPN in 2017.

	Drugs	Drug resistance rate (%)
ESBLs-KPN	Ertapenem	1.8
	Imipenem	2.8
	Piperacillin-tazobactam	8
	Amikacin	9.7
	Cefoperazone-sulbactam	21.7
	Tobramycin	27.8
	Gentamicin	40.9
	Fosfomycin	42.3
	Nitrofurantoin	48.3
	Levofloxacin	49.4
	Cefepime	50
	Ciprofloxacin	60.8
	Ceftazidime	63.1
	Aztreonam	73.9
	Cotrimoxazole	80.7
	Ampicillin-sulbactam	90.3
	Ceftriaxone	99.4
	Ampicillin	100

**Table 14:** Analysis of multiple drug resistance rates of CRE-KPN in 2017.

	Drugs	Drug resistance rate (%)
CRE-KPN	Cotrimoxazole	52.7
	Fosfomycin	60
	Amikacin	72.8
	Tobramycin	79
	Gentamicin	82.1
	Nitrofurantoin	96.3
	Ciprofloxacin	98.3
	Levofloxacin	98.3
	Aztreonam	98.6
	Cefepime	98.9
	Piperacillin-tazobactam	99.4
	Ampicillin	100
	Cefoperazone-sulbactam	100
	Ampicillin-sulbactam	100
	Ceftazidime	100
	Ceftriaxone	100
	Ertapenem	100
	Imipenem	100

**Table 15:** Analysis of multiple drug resistance rate of ESBLs-ECO in 2017.

	Drugs	Drug resistance rate (%)
ESBLs-ECO	Ertapenem	0
	Piperacillin-tazobactam	0
	Imipenem	0
	ASmikacin	2.2
	Nitrofurantoin	3
	Cefoperazone-sulbactam	6.4
	Fosfomycin	12.3
	Tobramycin	17.5
	Cefepime	32.9
	Gentamicin	41.1
	Ceftazidime	43.4
	Cotrimoxazole	53.9
	Aztreonam	66.3
	Ampicillin-sulbactam	66.8
	Levofloxacin	71.6
	Ciprofloxacin	75.8
	Ampicillin	99.3
	Ceftriaxone	99.5

**Table 16:** Analysis of multiple drug resistance rate of MDR-AB in 2017.

	Drugs	Drug resistance rate (%)
MDR-AB	Cotrimoxazole	74.7
	Amikacin	78.1
	Tobramycin	81.1
	Gentamicin	82.2
	Minocycline	84.3
	Ampicillin	100
	Piperacillin	100
	Piperacillin-tazobactam	100
	Ceftazidime	100
	Ceftriaxone	100
	Cefotaxime	100
	Cefepime	100
	Aztreonam	100

**Table 17:** Analysis of multiple drug resistance rate of MDR-PAE in 2017.

	Drugs	Drug resistance rate (%)
MDR-PAE	Polymyxin B	1.3
	Tobramycin	19.4
	Amikacin	46.6
	Gentamicin	69.2
	Cefoperazone-sulbactam	81
	Piperacillin/tazobactam	93.3
	Ciprofloxacin	97.7
	Piperacillin	99.3
	Aztreonam	99.3
	Cefepime	99.7
	Ceftazidime	100
	Imipenem	100
	Levofloxacin	100

**Table 18:** Analysis of multiple drug resistance rate of MRSA in 2017.

	Drugs	Drugresistance rate (%)
MRSA	Linezolid	0
	Vancomycin	0
	Nitrofurantoin	4.5
	Cotrimoxazole	10
	Rifampicin	28.9
	Gentamicin	39.1
	Levofloxacin	46.9
	Moxifloxacin	48.6
	Ciprofloxacin	51.1
	Clindamycin	51.7
	Tetracycline	52.5
	Erythromycin	61.1
	Oxacillin	100

**Table 19:** Analysis of multiple drug resistance rate of MRSA in 2018.

	Drugs	Drug resistance rate (%)
MRSA	Penicillin	100
	Oxacillin	100
	Erythromycin	74.3
	Clindamycin	69.1
	Tetracycline	38.4
	Ciprofloxacin	31.8
	Moxifloxacin	30.3
	Levofloxacin	28.3
	Cotrimoxazole	23.8
	Gentamicin	20.5
	Rifampicin	9.9
	Nitrofurantoin	1.3
	Linezolid	0
	Vancomycin	0
	Teicoplanin	0
	Tigecycline	0

**Table 20:** Analysis of multiple drug resistance rate of ESBLs in 2019.

	Drugs	Drug resistance rate (%)
ESBLs	Ceftriaxone	96.7
	Ampicillin-sulbactam	96.5
	Ciprofloxacin	67.2
	Aztreonam	64.7
	Levofloxacin	61.3
	Cotrimoxazole	56.2
	Ceftazidime	44.0
	Gentamicin	43.1
	Cefepime	36.2
	Tobramycin	21.5
	Nitrofurantoin	14.2
	Fosfomycin	13.6
	Cefoperazone-sulbactam	8.2
	Piperacillin-tazobactam	4.0
	Ertapenem	3.0
	Amikacin	2.7
	Imipenem	1.0

**Table 21:** Analysis of multiple drug resistance rate of CREs in 2019.

	Drugs	Drug resistance rate (%)
CREs	Ampicillin-sulbactam	97.1
	Imipenem	88.9
	Ceftriaxone	84.9
	Ertapenem	83.6
	Ceftazidime	82.7
	Nitrofurantoin	79.4
	Ciprofloxacin	78.9
	Aztreonam	77.3
	Levofloxacin	76.8
	Cefepime	75.8
	Piperacillin-tazobactam	74.5
	Cefoperazone-sulbactam	70.4
	Gentamicin	53.5
	Tobramycin	50.0
	Cotrimoxazole	43.3
	Amikacin	31.6
	Tigecycline	0.0

**Table 22:** Analysis of multiple drug resistance rate of MRSA in 2019.

	Drugs	Drug resistance rate (%)
MRSA	Oxacillin	100
	Penicillin	99.2
	Erythromycin	79.5
	Clindamycin	76.5
	Tetracycline	51.6
	Ciprofloxacin	31.5
	Moxifloxacin	29.5
	Levofloxacin	28.2
	Gentamicin	20.3
	Rifampicin	10.6
	Cotrimoxazole	6.1
	Nitrofurantoin	2.3
	Linezolid	0
	Vancomycin	0
	Teicoplanin	0
	Tigecycline	0

## Data Availability

The data used to support the findings of this study are available from the corresponding author upon request.
